# Supplementary material for: High Rate Performance Supercapacitors Based on N, O Co-Doped Hierarchical Porous Carbon Foams Synthesized via Chemical Blowing and Dual Templates
Source: Molecules. 2023 Oct 9;28(19):6994. doi: 10.3390/molecules28196994 (PMC10574032; doi:10.3390/molecules28196994)
Supplement: Supplementary file 1 [file molecules-28-06994-s001.zip › molecules-2578639-supplementary.pdf]

High rate performance supercapacitors based on N, O co-doped hierarchical porous carbon foams synthesized via chemical blowing and dual templates

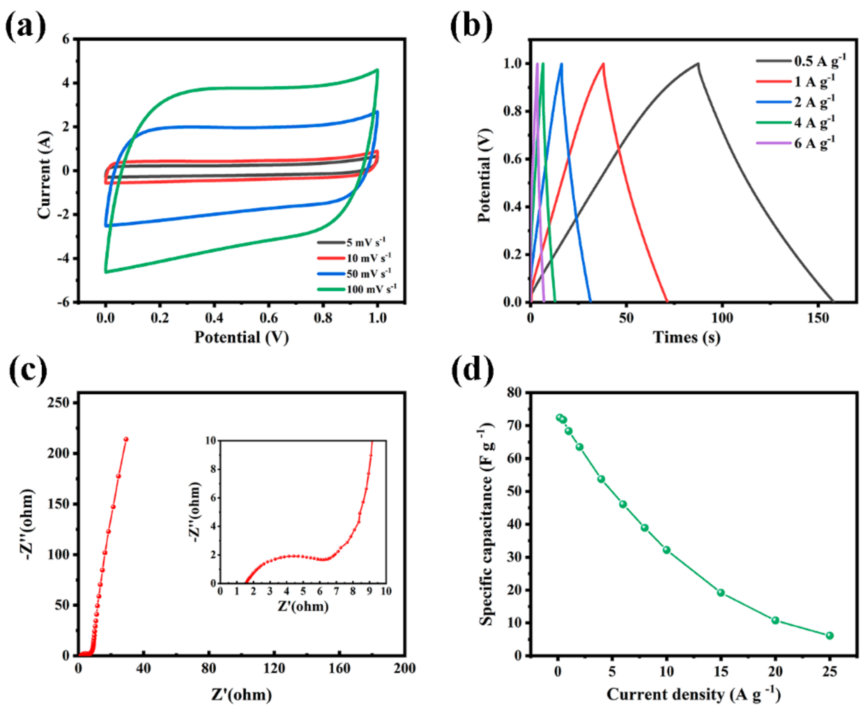

Figure S1. Capacitance performance of the commercial sample YP50F in a symmetric supercapacitor in 6 M KOH electrolyte with a mass loading of 2.7 mg in each electrode: (a) CV curve at different scanning rates, (b) GCD curve at different current densities, (c) EIS diagram and (d) rate performance.

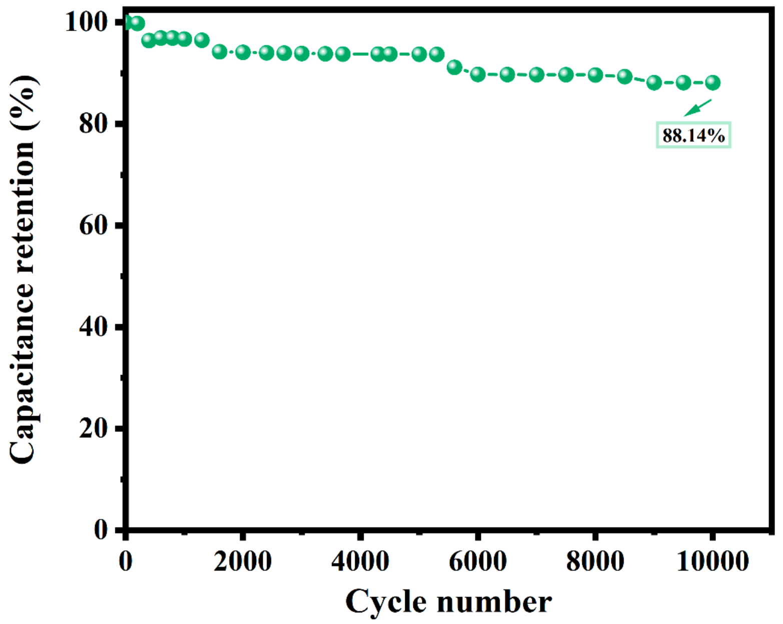

Figure S2 Long cycle performance of the symmetric supercapacitor based on CZnFe-5 at 2 A g^{-1} in 6 M KOH.
